# Distress in the care of people with chronic low back pain: insights from an ethnographic study

**DOI:** 10.3389/fsoc.2023.1281912

**Published:** 2023-11-16

**Authors:** Miriam Dillon, Rebecca E. Olson, Stefanie Plage, Maxi Miciak, Peter Window, Matthew Stewart, Anja Christoffersen, Simon Kilner, Natalie Barthel, Jenny Setchell

**Affiliations:** ^1^School of Social Science, University of Queensland, Brisbane, QLD, Australia; ^2^Physiotherapy Department, Royal Brisbane and Women’s Hospital, Brisbane, QLD, Australia; ^3^Faculty of Rehabilitation Medicine, University of Alberta, Edmonton, AB, Canada; ^4^Champion Health Agency, Brisbane, QLD, Australia; ^5^Psychology Department, Royal Brisbane and Women’s Hospital, Brisbane, QLD, Australia; ^6^Royal Brisbane and Women’s Hospital, Brisbane, QLD, Australia; ^7^School of Health and Rehabilitation Sciences, University of Queensland, Brisbane, QLD, Australia; ^8^Institute for Urban Indigenous Health, Brisbane, QLD, Australia

**Keywords:** chronic pain, sociology of emotions, distress, physiotherapy, health sociology, low back pain, affective assemblage

## Abstract

**Introduction:**

Distress is part of the experiences and care for people with chronic low back pain. However, distress is often pathologised and individualised; it is seen as a problem within the individual in pain and something to be downplayed, avoided, or fixed. To that end, we situate distress as a normal everyday relational experience circulating, affecting, moving in, through, and across bodies. Challenging practices that may amplify distress, we draw on the theorisation of affect as a relational assemblage to analyse physiotherapy clinical encounters in the care of people with chronic low back pain.

**Methods:**

Adopting a critical reflexive ethnographic approach, we analyse data from a qualitative project involving 15 ethnographic observations of patient-physiotherapist interactions and 6 collaborative dialogues between researchers and physiotherapists. We foreground conceptualisations of distress— and what they make (im)possible—to trace embodied assemblage formations and relationality when caring for people with chronic low back pain.

**Results:**

Our findings indicate that conceptualisation matters to the clinical entanglement, particularly how distress is recognised and navigated. Our study highlights how distress is both a lived experience and an affective relation—that both the physiotherapist and people with chronic low back pain experience distress and can be affected by and affect each other within clinical encounters.

**Discussion:**

Situated at the intersection of health sociology, sociology of emotions, and physiotherapy, our study offers a worked example of applying an affective assemblage theoretical framework to understanding emotionally imbued clinical interactions. Viewing physiotherapy care through an affective assemblage lens allows for recognising that life, pain, and distress are emerging, always in flux. Such an approach recognises that clinicians and patients experience distress; they are affected by and affect each other. It demands a more humanistic approach to care and helps move towards reconnecting the inseparable in clinical practice—emotion and reason, body and mind, carer and cared for.

## Introduction

Distress is part of pain experiences and care. However, it is often individualised and seen as pathology within the patient: a problem associated with poor prognostic factors and as something to be downplayed, avoided, or fixed (Dillon et al., Forthcoming). This article responds to calls to attend to the socioemotional complexities of pain and distress in clinical encounters (Bendelow, 2013; Dillon et al., Forthcoming). It draws on observations and relational theorisation^1^ to analyse and reconceptualise distress within clinical encounters related to the care of people with chronic low back pain (CLBP) to challenge individual pathology practices that can amplify distress.

Building on previous work (Dillon et al., Forthcoming), this article uses the theorisation of affect as a relational assemblage^2^ to explore who is distressed, how distress is expressed and responded to, and what distress does in clinical encounters. Addressing a critical gap in the literature, we argue that distress often needs to be normalised instead of pathologised in clinical settings. To that end, we situate distress as a normal everyday relational experience circulating, affecting, and moving in, through, and across bodies. Helping patients and clinicians to recognise and navigate experiences of distress may allow them to work productively and collaboratively to manage CLBP.

Positioned at the intersection of health sociology, the sociology of emotions, and physiotherapy, this study examines physiotherapy clinical encounters in CLBP care and argues that conceptualisation of distress matters in how it is recognised and navigated. In adopting a critical reflexive ethnographic approach, we draw on three tiers of data collection and analysis—observations of patient–physiotherapy interactions, collaborative dialogues with physiotherapists, and consultatory meetings with people with lived experience of CLBP—to investigate how distress circulates in clinical encounters. Overall, we foreground conceptualisations of distress—and what they make (im)possible—to trace embodied assemblage formations and relationality in CLBP care. We begin by situating distress in CLBP.

## Background and literature review

### Pain

Pain is ubiquitous in the human experience. CLBP persists or recurs for many years and is often enigmatic in its origins, pathophysiology, diagnosis, and management (Costa et al., 2022). Pain is a complex inter-relationship of biological, psychological, spiritual, contextual, experiential, social, cultural, material, and other elements (Mescouto et al., 2023). Therefore, particularly in its chronic form, pain challenges traditional biomedical approaches, as it complicates the borders of mind and body, objective and subjective, and physical and emotional (Borkan, 1993). The International Association for the Study of Pain’s (2020) widely recognised definition stipulates that pain is both a “sensory and emotional experience associated with actual or potential tissue damage” (p. 5). Despite this, the enactment of pain care tends to be biomedically focused with an overemphasis on the sensory and physical aspects in both diagnosis and treatment (Bendelow, 2013; Mescouto et al., 2022a; Dillon et al., 2023). Such dualism works to separate the physical from the emotional aspects of pain, enforce rational and objective approaches, and attribute “single symptoms to single causes” (Bendelow, 2013, p. 456). Yet, experiences of pain are subjective, value-laden, and involve the attribution of meaning through experience, making pain difficult to objectively define or quantify (Bendelow, 2013; Ahmed, 2014). Traditional biomedical and narrow psychological approaches to pain research and care strive for objectivity, with limited consideration of its social, cultural, or power dimensions (Mescouto et al., 2022a). They limit clinicians’ abilities to see patients as people living in a complex and dynamic world with unique lives, histories, capacities, and needs who are affected by a range of sociocultural-material mediators (Gibson et al., 2023). Within the field of chronic pain, calls have been made for more engagement with social and emotional influences in understanding and managing pain to help address its complexity (Bendelow, 2013; Dillon et al., Forthcoming).

In the following literature review, we explicate why it is necessary to unpack the socioemotional entanglements circulating in the care of people living with pain. In particular, we focus on emotions like distress within physiotherapy clinical encounters. We begin by exploring emotions and distress and how they are currently understood in society more broadly. We then attend to how emotions, distress, and care are situated within experiences of CLBP and physiotherapy treatment. In doing so, we highlight why distress cannot be avoided in physiotherapy care and how current approaches are insufficient at meeting the needs of both patients and clinicians.

### Emotions and distress

Emotions are complex, much like pain. They are understood, experienced, and expressed differently in various cultural, social, organisational, and political settings. Emotions can be broadly understood as physiological, cognitive, relational, and affective phenomena: embodied sensations and expressions that underpin reason and motivate people to (inter) act, mediated by cultural and social expectations and labels (Jasper, 2018). However, historical understandings of emotions continue to pervade Western thought in science, philosophy, and everyday life. These understandings primarily situate emotions in dualistic, disembodied, and hierarchical ways—separating emotion and reason, body and mind (Ahmed, 2014; Wettergren, 2019). In addition, the bifurcation of emotion and reason is the ongoing treatment of emotion with suspicion rather than as helpful, functional, or wise (Jasper, 2018). Emotions are viewed as problematic, residing within an individual, welling up from within, affecting one’s judgement, or causing one to be reactive rather than active (Ahmed, 2014; Jasper, 2018). Although emotion management is often required (Hochschild, 2012), emotions are part of what it is to be human, permeating all human experiences and interactions. Therefore, they cannot and should not be avoided.

Distress is an equivocal term, often representing a constellation of emotions and experiences assigned negative valance. In our previous work, we argued that the historical but ongoing dualistic treatment of emotion and the body may explain why distress is pathologised and individualised in CLBP care (Dillon et al., Forthcoming). Furthermore, understandings of distress have shifted from being approached as an experience embedded in sociomaterial conditions to an abstracted medical pathology (Dillon et al., Forthcoming). This may, in part, be a consequence of rising neoliberal or capitalistic political agendas in Western societies, which situate human suffering or distress as based on “faulty minds and brains rather than on harmful social, political and work environments” (Davies, 2022, p. 8). Davies (2022) suggested that the marketisation of mental health has depleted human suffering or distress of its “deeper meaning, and purpose, consequently, our distress is no longer seen as a vital call to change or as anything potentially transformative or instructive” (p. 8), but as something that targeted consumption (such as diet or fashion) can fix. Furthermore, the evolution of the Diagnostic and Statistical Manual (DSM) is argued by some to wrongly pathologise and medicalise much of our everyday distress, rebranding often normal and reasonable human emotional experiences as psychiatric illness (Davies, 2022).

Historical and contemporary understandings of emotions and distress go some way to explaining why distress is often problematically situated in CLBP care.

### Distress and CLBP care

A small but growing body of research has focused on the therapeutic relationship between physiotherapists and patients, including within pain care. Positive relationships have been associated with improvements in patients’ pain intensity (Fuentes et al., 2014), physical function (Ferreira et al., 2013), and mood (Alodaibi et al., 2021). Therefore, interactions with healthcare professionals like physiotherapists are instrumental to individuals’ experiences of pain treatment and management. Yet, despite the growing body of scholarship implicating connections between pain and distress, little attention beyond acknowledgement has been given to distress in CLBP care, particularly within clinical encounters (Dillon et al., Forthcoming). This may be one (of many) reasons why patients often report having disappointing relationships with healthcare professionals, articulating that they feel unheard, uncared for, or not taken seriously (Costa et al., 2022; Mescouto et al., 2023).

Research on therapeutic relationships tends to focus on developing strong relationships, with little attention paid to any tensions or ruptures (Miciak and Rossettini, 2022). Physiotherapists often avoid relational tensions, misjudge the cause or fail to recognise or acknowledge their own role in therapeutic relationship breakdowns (Miciak and Rossettini, 2022). However, relational ruptures and tensions are inevitable and normal (Gelso and Kline, 2019). If navigated well, they can be opportunities to strengthen relationships and improve patient outcomes (Miciak and Rossettini, 2022). Given that distress is a potential source of relational tension, being able to understand and appropriately respond to distressing situations is paramount to sustaining the therapeutic relationship.

While the primary focus in contemporary research is often on patients’ distress in clinical encounters, emergent work has highlighted clinicians’ distress as well. Notably, clinicians who struggle to navigate their own and their patients’ distress have been found to be at higher risk of clinician burnout and detachment in multidisciplinary pain clinics (Ashton-James et al., 2021). As part of multidisciplinary pain clinics and elsewhere, physiotherapists regularly encounter patients who experience distress in their day-to-day work (McGrath et al., 2023), and they may similarly find such encounters emotionally exhausting, or they invoke their own empathetic distress (McGrath et al., 2022). Further research grounded in sociological theory would assist physiotherapists in understanding, recognising, and navigating distress, including in pain care.

### Physiotherapy and CLBP care

Physiotherapy’s traditional emphasis on physical and biomedical aspects of care situates the body-as-machine (Nicholls and Gibson, 2010), which can constrain physiotherapists’ ability to navigate emotions and distress within clinical encounters. Historically, physiotherapy’s focus on function and dysfunction within the mechanical body was useful to differentiate it from other health disciplines and give it legitimacy and place within the healthcare industry (Nicholls and Gibson, 2010; Schwab et al., 2023). Originally a profession dominated by women, physiotherapy first emerged as a therapeutic massage practice and later rose to prominence as a unified profession during the First World War as part of rehabilitation efforts for war veterans (Linker, 2005). Early approaches contributed to a decontextualisation of the body, reducing it to its mechanical functions: something to be “fixed” (Gibson et al., 2018; Schwab et al., 2023). Many aspects of these historical practices continue to define physiotherapy today, and this approach leaves little space for the consideration of distress.

Critical physiotherapy scholars have recently argued that physiotherapists need more than technical competence and must engage in relational ways of being with their patients (Kleiner et al., 2023). The implementation of broader approaches like the biopsychosocial model strives to change how physiotherapists understand the multidimensional aspects of pain and approaches to care (Mescouto et al., 2020). However, these approaches have not yet succeeded in applying more integrated care (or a nuanced understanding of pain) to clinical practice, often applied in a fragmented fashion, with biomedical and behavioural aspects of care overemphasised (Mescouto et al., 2020). Lack of philosophical/theoretical clarity and failure to consider other elements, such as power, have been suggested as possible reasons for the limited success of the biopsychosocial model in clinical practice (Mescouto et al., 2023). This is also reinforced by contemporary physiotherapy education and training, where competency in procedural knowledge is considered an essential skill, while critical reflexivity (Schwab et al., 2023) and attention to complexity are not.

Current physiotherapy knowledge and practices limit understanding of people’s whole pain experience—prioritising physical and limited psychological aspects, artificially separating these aspects from others, such as emotions. Objective knowledge is prioritised as tangible in language and clinical tests, sidelining less tangible knowledge, such as embodied, affective, and non-verbal knowledge. Not only do contemporary approaches fail to adequately account for patients’ emotions, they overlook physiotherapists’ emotions and how these emotions intersect. Few opportunities are provided for physiotherapists to recognise their own or patients’ distress. This may exacerbate their individual or mutual distress, amplify patients’ pain experiences, or contribute to their poor engagement in care. In the following section, we outline our critical, sociocultural, affective, and relational approaches.

## Theoretical framework

How distress is conceptualised matters (Dillon et al., Forthcoming). The approach to distress shapes what one sees, avoids, and how one (inter) acts (Olson et al., 2020). In this study, we build on sociological theories of emotions to examine how distress is conceptualised and what it does within physiotherapy clinical encounters. Emotion is a contested and complex concept (Olson et al., 2020). While classical psychology may alert physiotherapists to a patient’s distress through observation of physical changes loosely associated with emotion, it frames distress as physiological, universally expressed/experienced, and in individualised, dualistic, reductionist ways—something to be avoided or fixed (Dillon et al., Forthcoming). Symbolic interactionist approaches expand on physiological framings to foreground the influence of social and organisational culture in shaping expectations of what emotions are and how, when, or where they should be expressed to conceptualise emotions as co-constructed within social contexts (Hochschild, 2012). Within physiotherapy, these social interactions are often guided by social scripts^3^, such as an overly positive physiotherapist encouraging following the “cheer” script (Setchell et al., 2019) or the biomedical script (Dillon et al., 2023). Symbolic interactionism shifts from the idea of emotions as existing in one person towards the recognition of emotions as interpersonal and plural. Consequently, symbolic interactionism fosters acknowledgement that physiotherapists may also experience distress and highlights how physiotherapist and patients may manage their emotions or shape how they express them in order to comply with the “feeling rules” of the clinical context. However, this approach gives primacy to language and culture and, for our analysis, does not go far enough in recognising emotions as affective and relational (lowlighting the embodied, non-verbal, and pre-conscious aspects of emotions).

We prioritise a reconceptualisation of distress that foregrounds how distressed bodies (patients-physiotherapists-and beyond) relate to “human, non-human, self and the other” (Dragojlovic and Broom, 2018, p. 3). Taking a critical, sociocultural, affective, and relational approach, we reconceptualise distress as an affective assemblage of bodies (human and non-human), discourses, practises, and performances (Dragojlovic and Broom, 2018). Specifically, we draw from Ahmed’s relational and affective theorisation of emotion and DeleuzoGuattarian assemblage theory.

Ahmed (2014) focused on relations between emotions, language, and cultural and political discourses and bodies, suggesting that emotions can be entangled in acts of speech, felt sensations, and objects. Shifting from individually experienced emotions, Ahmed (2014) suggested emotions *do* things, circulating between and affecting bodies. According to Ahmed (2014), emotions take the shape of the contact or the orientation a subject has with another subject or object—material and immaterial. This contact involves the subject and histories that come before the subject and intensities. Ahmed (2014) highlighted how emotions are relational: “they involve (re) actions or relations of towardness or awayness in relation to such objects” (p. 8). Emotions are not just about orientation or movement but attachments—what connects us to something or someone (or not). Furthermore, emotions accumulate affective value due to time and repetition of contact, influencing intensities and interactions. This is critically important when we consider clinical encounters. How a physiotherapist interacts with a patient is influenced by many things, which may offer opportunities (or not) for them to connect with their patients. Within clinical encounters, the same can be said for patients.

DeleuzoGuattarian ontology sees human bodies and all other material, social, and abstract entities as relational (Fox and Alldred, 2015). Affects—active forces that shape an individual or thing’s capacity to “affect or be affected” (Fox, 2015, p. 306)—constitute an assemblage. Emotions are one form of affect “part of a continuum of affectivity that links human bodies to their physical and social environment” (Fox, 2015, p. 302). Assemblage relations emerge in unpredictable ways around actions and events, always in flux or operating as machines that do or produce things (Fox and Alldred, 2015).

For example, in a clinical interaction between a health professional and patient, there are many sources of affective flow: two human bodies, subjectivities, multiple sources of knowledge, a clinic room, a team, a hospital, a health system, furniture, social positionings, technology, lives outside the clinic, histories/past experiences, experiences of marginalisation and stigmatisation, and so on. The relations among all these things form the assemblage, which is in a dynamic state of flux with various elements coming to the fore, dropping away, or adding on at different times. Countering dualistic conceptualisations, we strive to make no separation in our conceptualisation of assemblages between “conscious and unconscious, intentional and non-intentional, mind and the body, human and non-human” (Dragojlovic and Broom, 2018, p. 5). We acknowledge that these elements of the clinical assemblage are different. However, we attempted to avoid making rigid distinctions between these elements as they are all affective and connect with distress. This reconceptualisation shifts away from imaginings of distress as individually experienced, dualistic, and static towards a recognition of distress as dynamic: subject to unstable forces that can emerge in unpredictable ways around actions and events.

A critical, sociocultural, affective, and relational lens serves as the theoretical foundation for this study, providing a broader perspective for attending to individuals in pain and their physiotherapists within fluid physical and sociocultural worlds. This allows us to view distress as more than a pathology: an often-normal response to experiencing and striving to treat pain. To explore the assemblage, we take it apart to examine its elements and the nature of the relations comprising it (Dragojlovic and Broom, 2018). Our focus is on the lived bodies within physiotherapy clinical encounters to investigate who is distressed and how distress is expressed, recognised, and responded to. We use the three theories of emotions summarised above as lenses for analysing clinical encounters and to demonstrate what conceptualisations of distress *do*.

## Methodology

This article draws on data from a larger seven-month project that explored the distress–pain–assemblage in physiotherapy CLBP care. For this study, we sought to explore how distress circulates and what distress—and conceptualisations of distress—*do* within clinical encounters between physiotherapists and patients with CLBP. As we need our methods to attend to the specificities of clinical encounters and the complexity of distress, including embodied and affective components, we took a critical reflexive ethnographic approach (Davies, 2007). Data were produced ethnographically, through observations of patient–physiotherapy interactions, and reflexively, through collaborative dialogues with physiotherapists. Ethics approval was provided by the institutional Human Research Ethics Committee: HREC/2021/QRBW/77069. This research was conducted in accordance with the Australian National Statement on Ethical Conduct in Research (AVCC, 2018).

### Research context and participants

The study was conducted in an advanced practice physiotherapy-led musculoskeletal service within a publicly funded hospital in an urban area in Australia. At the clinic, people with various presentations on waiting lists for surgical review are assessed by advanced practice musculoskeletal physiotherapists known as clinical leaders, and a pathway of care is determined. This may include referral to a medical specialist where indicated, or if they are likely to benefit from non-surgical intervention, a referral to multidisciplinary care that may include any or a combination of physiotherapy, psychology, occupational therapy, pharmacy, and/or dietetics based on the patient’s needs.

Participants in the study included as follows: (i) 15 adults with CLBP referred to the service, (ii) 14 physiotherapy clinicians who worked with them, including clinical leaders and treating physiotherapists (some worked across both roles and locations). Patients were eligible if their clinician had determined that they experienced CLBP and were 18 years or older. Purposeful sampling was applied to include a diverse range in terms of age, gender, symptom duration, and pain intensity. All physiotherapists working within the clinic were considered eligible and invited to participate. Participants were excluded if they were patients but did not experience CLBP or were not 18 years or older or if they were clinicians but not physiotherapists.

### Data production

Two forms of data were collected between June 2022 and December 2022.

Ethnographic observations of fifteen 30–90 min physiotherapy–patient interactions were produced to capture the complexity of clinical encounters. These were completed by a trained observer, the lead author. She wrote reflexive fieldnotes while sitting in the room during consultations, describing the clinical encounter, focusing on who was distressed, how it was expressed and navigated, and how it circulated and affected bodies, along with descriptions of the sensations, thoughts, feelings, and intensities experienced by the observer.

Clinician dialogues consisting of six 2-h reflexive discussions with physiotherapy-participants—three at each location at two-month intervals during the study—supplemented observations. All physiotherapists working within the clinic were invited to participate. Dialogues were facilitated by three members of the research team—the lead authors—MD, JS, and PW. Researchers shared observation excerpts and facilitated reflexive exercises during the dialogues with the aim of gaining insight into how clinicians understood and experienced distress and the factors that influenced their distress. Emerging study findings from iterative analysis in research team analysis meetings and our discussions with consumer panels (see below) were also shared to encourage further reflexivity. Due to scheduling complexities, dialogues were repeated two times: 11–14 clinicians attended per dialogue session. Dialogues were audio-recorded and transcribed verbatim. Gender-neutral pseudonyms were used in field notes and transcriptions.

### Data analysis

Analysis was conducted in three iterative collaborative spaces to engage a variety of perspectives from clinicians, consumers, and researchers.

Research team analysis meetings (*n* = 7): The research team met monthly throughout the course of the project. The team was comprised of two consumer experts—people with lived experience of CLBP (one of whom had been a recipient of care within the clinic), two physiotherapy researchers with clinical experience, two physiotherapy researchers with psychology and sociology training, emotions and health sociologist, a clinical psychologist, and a clinical physiotherapist. Two of the physiotherapists were working at the study site while data was generated. Selected field notes of observations and excerpts or key findings from clinician dialogues were shared with the research team and analysed considering who was distressed, expressions of distress, factors influencing distress, and how distress was conceptualised and navigated.

Consumer panels (n = 2): We conducted two consumer panels, one mid-way through the project and one at the end. Panels comprised six people with lived experience of CLBP, two of whom had received care through the clinic and four who received care at other healthcare facilities, public and private. All consumers had received care from multiple healthcare professionals as part of their CLBP journeys. Panel members were advisors, not study participants, and provided feedback on the study design and data analysis. Excerpts from observation fieldnotes and clinician dialogues were shared with panel members as part of the data analysis process.

Clinician dialogues (n = 6), described above, formed part of both the data generation and analysis process, allowing insight into clinicians’ experiences and reflexive interpretations of data.

Building on the analysis achieved through research team analysis meetings, consumer panels, and clinician dialogues, the core research team (MD, RO, and SP) subsequently analysed the data post-paradigmatically. That is, they abductively (Tavory and Timmermans, 2014) foregrounded diverging theoretical paradigms for conceptualising emotions in social life during the latter phases of analysis to attend to what differing conceptualisations of emotions do within clinical encounters (Olson et al., 2020).

The first author, MD, led the analysis throughout, whose social positioning undoubtedly shaped the analysis—she is both a physiotherapist and a sociology researcher. Recognising that her training, values, interests, and past experiences were part of the research, MD engaged in critical reflexivity (Thille et al., 2018) throughout the research process to help understand the assumptions that may have influenced the analysis and findings. She included reflexivity in ethnographic fieldnotes and kept a reflexive diary throughout the project. The diverse positions and perspectives of the interdisciplinary research team, as well as people who had experience working or receiving care within the clinic and others who were external to the clinic, also provided balance. However, working as an insider and outsider meant the first author found data generation and analysis to be a confronting experience. While it helped to recognise tendencies to be task-focused or habitual/scripted practices that may have resulted in patients feeling unheard, dismissed, or invalidated, shame and guilt were inevitable in recognising the limitations of her own therapeutic approach and the potential impact of her own distress on patients. It also gave a new appreciation for different ways of knowing through embodied sensations and affect, such as embodied tension because of the therapist’s own distress—because of time pressures, back-to-back complex patients, life stress, or uncertainty about how to help a person.

Next, we present our findings, focusing largely on what occurred relationally within clinical encounters. Non-human elements within the affective assemblage are included at times but foregrounded in more detail elsewhere. Of note, findings from ethnographic observations feature prominently in the results, with analytic insight from clinician dialogues and patient consultatory panels supporting our interpretations of the results and discussion.

## Results

Distress was present in all clinical encounters. Patients’ expressions of distress were overt, such as body language, actions, direct use of words, sweating, and red face; and subtle, such as repetition of words, story, humour to conceal discomfort, and silence. Moreover, it was entangled with physiotherapists’ distress, though this was not always recognised. Through our analysis, we found that physiotherapists responded to patients’ (c) overt distress in the following three ways: (1) avoiding and trying to manage it, following various scripts, (2) not acknowledging or recognising it, and (3) recognising and responding relationally.

In the following sections, we draw on the theorisation of affect as a relational assemblage to explore who is distressed, how distress is expressed and responded to, and what distress does in clinical encounters. Imposing an organisation on this affective assemblage, we start with physiotherapists’ most prevalent approach: avoiding and managing. Overall, we attend to what distress and (non) responses to it did within the affective assemblage—while also demonstrating that all physiotherapy clinical encounters undertaken with individuals with CLBP are affective entanglements and require broader conceptualisations of distress and relational approaches to care.

### Downplaying, managing, and avoiding

In many of the observations, physiotherapists were noted to be following varied scripts related to distress: cheer, biomedical, and protocol (explained below). Here, we explicate some of the various ways patients expressed their distress, the ways physiotherapists avoided or tried to manage the distress, and what this did within clinical encounters.

#### AlexPT^4^ and Taylor—cheer script

The “cheer script” was observed very distinctly throughout two of the 15 encounters in response to patients’ sad words, stories, and/or body language. For example, in Taylor’s third encounter with AlexPT, Taylor’s distress was easily identified by AlexPT in their body language: sitting slumped on the edge of the bed, looking to the ground, a sad facial expression, a soft, often mumbling, flat, monotone voice, and minimalist answers. AlexPT was observed noticing this and responding by attempting to cheer Taylor up. AlexPT made jokes and laughed, but such positivity potentially invalidated Taylor’s distress and pain. For example, when AlexPT asked Taylor to perform movements of their spine, Taylor shared, “I hate this one.” AlexPT asked, “What do not you like about it?” Taylor replied, “It’s sore … my love handles get in the way.” AlexPT laughed loudly (sounding a bit forced) and asked brightly, “Feel stuck?” Taylor stood up after the movement and rubbed their back, replying, “Yeah, it sets off the pain in this side of my back.” AlexPT laughed again and moved on without acknowledging or attending to Taylor’s implicit distress.

Later, AlexPT reassessed Taylor’s home exercises and said brightly, “That’s actually heaps better than the last time you came in, even being able to progress it to being able to do it without sitting all the way down is really, really good.” This cheer and encouragement contrasted with Taylor’s body language and repeated statements about the lack of improvement in their pain.

Physiological presentations of distress can helpfully alert clinicians to a patient’s distress (Dillon et al., Forthcoming). Taylor’s body language, soft monotone voice, and short answers may have alerted AlexPT to Taylor’s distress, but AlexPT’s *cheer* (Setchell et al., 2019) *response* aligns with physiological conceptualisations of distress as pathology. It implies distress is something to be downplayed or eliminated^5^ rather than a productive opportunity to connect and understand their whole pain experience (Dillon et al., Forthcoming). It suggests an interpretation of emotions as irrelevant to the clinical interaction, something to be managed through—in this case—laughter and positivity (McNaughton, 2013). The impetus behind attempts to manage or avoid distress was the focus of discussion in clinician dialogues. Physiotherapists told us that difficult emotions, such as distress, are sometimes avoided for fear of opening “the can of worms” and not being able to “contain the can,” indicating fear or lack of training in navigating challenging emotions. Some physiotherapists asserted it was not within physiotherapy’s scope of practice and should be avoided. It may also be related to an inability to recognise and sit with their own discomfort with a patient who is upset (Setchell et al., 2019). Thus, a lack of understanding of how to navigate distress along with organisational or professional normative expectations may have informed AlexPT’s attempts to manage Taylor’s distress through cheer.

AlexPT’s cheer response—evident through joking, laughing, positive encouragement, bright tone of voice, and smiling—contrasted with their body language immediately following the consult. They were “red and looked tired” and expressed how difficult the encounter was, which was an acknowledgement of their own distress. Viewing distress through a symbolic interactionist lens recognises that clinicians also experience distress, but sociocultural forces or “feeling rules” dictate where, when, and how distress or emotions are expressed (Hochschild, 2012). As Hochschild (2012) asserts, individuals may modify their feelings to comply with organisational and cultural forces, suggesting that AlexPT may have been “surface acting”^6^ in an attempt to maintain a positive cheerful response and avoid/move around Taylor’s distress to comply with institutional and cultural expectations that challenging emotions should be downplayed. AlexPT’s own distress—the toll that managing Taylor’s emotions took—also foregrounds the relationality of the distress affective assemblage.

#### RileyCL^7^ and Finlay—biomedical script

While some physiotherapists responded to distress by downplaying and attempting to manage it through cheer, others conformed to biomedical scripted or checklist approaches to care. Finlay’s first encounter with RileyCL offers an example. Finlay’s distress was clear from the moment they entered the clinic and continued, fluctuating in intensity, throughout the encounter. It was evident in their body language, tone of voice, actions, and words but not acknowledged or foregrounded by RileyCL. RileyCL’s biomedical scripted approach enabled them to do this by placing them in a position of power, controlling the encounter and prioritising the physical aspects of Finlay’s pain.

Finlay was observed in the waiting room by the ethnographer moving around a lot and explained to the ethnographer with a stressed tone of voice how they were “in agony” and “so nervous” about the appointment. Within the encounter, Finlay’s body language conveyed distress. They sat at the edge of their chair, bracing their hands on their thighs. They were observed fidgeting and shuffling their feet while RileyCL spoke. Later, Finlay started visibly sweating and took a fan from their handbag and began fanning their face. They said, sounding distressed, “I’m going through really bad menopause, so please do not, I get so hot” RileyCL laughed^8^ and moved on. RileyCL, in contrast, was confident and assertive, sitting composed, and upright in their chair. RileyCL, often typed as they spoke, was tasked with focused and prioritised facts about the physical elements of Finlay’s pain, ignoring or quickly moving on from Finlay’s implicit presentation of feelings.

RileyCL controlled the encounter by speeding up and slowing down their questions or interrupting and changing direction, dependent upon the information they deemed to be important—the biomedical/physical elements. For example, when exploring Finlay’s level of function, RileyCL interrupted Finlay and asked, “So what else are you doing, other than walking for an hour?” Finlay hesitated before replying, “Ah, um…” Without receiving a reply, RileyCL quickly asked, “How do you spend your time? You have grandkids? Your daughter?” Finlay sat down slowly and replied, “Mum takes a lot of my time up.” Finlay continued to explain in a sad tone of voice how they could no longer physically care for their mother. RileyCL interrupted them again and said, “I’m more trying to get an idea of what your day looks like, Finlay.” Finlay replied “Oh right, so worrying about mum all day, every day. Crying. It’s full of anxiety… *It’s really hard. It’s really, really hard*.” Finlay emphasised their final words with a sad tone and was observed squinting their face. Despite Finlay’s (now) overt expressions of distress, RileyCL again quickly moved on and summarised only their patient’s physical ailments.

This biomedical scripted approach lends itself to paternalistic practice where the physiotherapist holds more power within the encounter, which is known to negatively impact the therapeutic alliance and patients’ engagement with physiotherapy (Mescouto et al., 2022b). It positions the clinician as the expert on the patient’s pain experience rather than a partnership of reciprocal and shared exploration. We noted this positioning throughout the observation, evident in how RileyCL controlled the encounter. It was also particularly notable at the end of the observation when RileyCL provided Finlay with a diagnosis and recommendations for treatment, speaking very quickly and providing very little opportunity for Finlay to comment or ask questions. RileyCL began this by explaining why they believed Finlay did not have nerve pain and why their scan results were not concerning. Finlay sat upright, perched at the front of their chair, looking intently at RileyCL, and responded hesitantly and slowly, saying, “okaaaay.” RileyCL carried on, speaking very fast, explaining that Margert had spinal arthropathy. Finlay interrupted and asked, “Is that where I was really sore?” RileyCL agreed; however, Finlay was not convinced and asked, “But what about when I am bending or trying to pick something up, so how come it’s always there?” RileyCL speaking very quickly again (the ethnographer noted their struggle in keeping up), provided a brief explanation, then continued to explain why Finlay did not need surgery. Giving Finlay limited opportunity to ask questions, RileyCL continued quickly providing treatment recommendations, “relaxation things, some specific control exercises, turning off some overactive muscles and turning on others.” RileyCL suggested psychology for “pain management, relaxation techniques, body scanning progressive muscle relaxation techniques to help manage pain.”

In prioritising a biomedical script, RileyCL detected distress but viewed emotions and distress as a stimulus–response irrelevant to the mind–body dualist biomedical encounter. In doing so, RileyCL missed the complexity of Finlay’s life experience, context, and emotions as other factors contributing to their distress and pain, which may negatively impact future engagement with treatment and its effectiveness. This approach also misses the relationality of emotions, lowlighting both the patient’s and clinician’s affect and comfort. RileyCL’s avoidance and task-focused approach may have exacerbated Finlay’s distress (and potentially their pain).

In clinician dialogues, physiotherapists identified with RileyCL’s approach, saying they were confident and comfortable when attending to the physical aspects of care. They described often feeling uncertain whether they should attend to emotions and distress. Some felt distress was something they would not be able to change and should focus on the physical elements they may be able to influence. Like the cheer script, some feared opening a “can of worms” and not being able to navigate it or contain it. Others suggested previous attempts to attend to distress going badly, for example, patients walking out of the appointment or failing to return to their next appointment and fear of similar reactions happening again.

However, this distress was not contained to the individual or separate from the lived reality of people experiencing CLBP; the physiotherapist’s avoidance of emotion was said to have a relational impact on the patient. In consumer panels, members discussed how physiotherapists’ task-focused approach could result in patients feeling misunderstood, invalidated, stigmatised, and not believed. One consumer suggested that physiotherapists who incorporate checklist biomedical approaches will never understand the person or get to the bottom of how to effectively work with people or successfully gain someone’s trust: “Clinicians need to understand that it’s their jobs but our lives.”

#### FrankiePT and Blair—protocol script

The biomedical scripted approach was prevalent, followed by almost all physiotherapists. However, not all physiotherapists remained composed and confident like RileyCL throughout. Many appeared uncomfortable when patients expressed distress, particularly when it was overtly and/or forcefully expressed in patients’ words and body language. For example, in Blair’s fourth encounter with FrankiePT, distress was evident in both from the onset, circulating and fluctuating throughout the encounter. FrankiePT’s distress was observed prior to the encounter; they “appeared flustered” and stated they were running late and feeling very tired, having attended a course all weekend. This was compounded by an email from the specialist physiotherapist managing Blair’s case, which FrankiePT perceived as criticising their treatment approach and directing a particular physical approach, which they tried in the session prior to the observed appointment, unconvinced it was appropriate. Blair also appeared distressed before the encounter, observed appearing “uptight,” with a “very shallow breathing pattern” and “elevated shoulders.”

Avoiding Blair’s evident unease, FrankiePT began by asking Blair to complete a questionnaire (a patient-reported outcome measure) and walked back out of the room. FrankiePT returned a few minutes later, and Blair immediately expressed their frustration. Turning red, Blair threw the questionnaire to FrankiePT, telling them the form was stupid. Sounding frustrated, they exclaimed, “Half of the questions I do not know, I do not agree with the questions. You persist with pain; you get on with it. You do things because you have to, you do not have a choice.” FrankiePT maintained what could be considered a formal demeanour, sat upright at the front of their chair, and did not acknowledge Blair’s distress. They simply replied, “Yeah ok.” FrankiePT then hesitated and put the questionnaire aside to ask, “How are you going?” Blair quickly replied, “That exercise did not help, it actually increased the pain.” FrankiePT potentially attempting to understand and tune in to Blair’s response, but not acknowledging Blair’s distress, immediately replied, “Tell me about the pain.” Blair explained how they struggled to get through the week due to increased pain, which impacted their ability to work. FrankiePT nodded as Blair spoke, with a serious facial expression, pursing their lips and maintaining eye contact.

FrankiePT, already distressed themselves, was confronted with Blair’s dissatisfaction. Blair’s response appeared to amplify their distress and possibly affected their capacity to attend to Blair’s distress or flexibly adjust or change direction when their original plan for the session was disrupted. FrankiePT looked uncomfortable but attempted to maintain a composed and formal Blair’s emotionally imbued response and moving on.^9^ Blair was reasonably distressed; their pain was escalating and impacting their capacity to function, with implications for work, compounded by being asked to complete a questionnaire that made no sense to them and with no explanation or help in completing it.

Distress may be generated or increased within clinical encounters by how clinicians ask questions and the perceived intent behind the questions or assumptions clinicians might make. For example, when FrankiePT asked Blair about their work, Blair explained, “it’s really affecting my ability to work.” FrankiePT asked about work hours, days, breaks, and shift patterns. Blair’s tone became more defensive and frustrated sounding. FrankiePT said, “still doing all those things?” (tasks Blair had described at work). Blair said firmly, “yes because I need a job.” In consumer panels, people with lived experience suggested physiotherapist’s questions, when gathering important information, can be construed as judgemental, condescending, or making assumptions about a person, causing them more distress. Some said they had similar experiences to Blair’s, feeling clinicians often assume pain is okay if you can push through, not recognising the impact this may have on other aspects of people’s lives or the distress this causes. It is possible that Blair felt judged by these questions, becoming more frustrated. We interpret their repeated explanations of how bad their pain was in the encounter as likely indicating that they felt unheard.

More than a physiological presentation, more than an inappropriate emotion to be managed, this encounter demonstrates the dynamic, circulating, and fluctuating nature of the pain-distress-assemblage. FrankiePT and Blair, in affecting each other, appeared to become more distressed as the encounter progressed. Like AlexPT, FrankiePT expressed relief when Blair left, saying how difficult the encounter was. This observation demonstrates the emotional labour FrankiePT was doing to manage their own distress. Knowing Blair was distressed but possibly not knowing how to navigate it other than maintaining a formal demeanour, FrankiePT followed a protocol script: moving around and avoiding Blair’s distress to attend to the questionnaire and questions on their checklist.

Physiotherapist participants told us how patient distress can evoke feelings of helplessness, hopelessness, and uncertainty. Some also suggested that their distress within clinical encounters was also influenced by workplace culture-based expectations, which meant that they feared making a mistake, not meeting organisational expectations, or coming up against conflicting ideas within the team of what treatment should involve. This encounter demonstrates that emotions and distress are physiological and socioculturally relevant but also relational and shared. Boarder conceptualisations of distress are required.

### Not acknowledged or not recognised

In some observations, distress was only subtly evident in patients’ words but not recognised by physiotherapists. MorganPT and Parker’s initial clinical encounter offers an example.

#### MorganPT and Parker

MorganPT’s distress was evident to the ethnographer from the outset. MorganPT stated that one of their children was sick and had to do extra drop-off to childcare, so he was running late and was very tired. Despite this, the encounter began pleasantly with moments of shared laughter, but it was not long before signs of Parker’s distress emerged, evident in their words as they recalled an incident from 10 months ago when they bumped into a door frame at the same time as their son-in-law was in hospital. Parker described how, an hour later, they were unable to move. “I had severe pain, could not breathe, or sit… I went to the GP and got medication for muscle spasm. It was horrendous pain and took a few days to settle even with the medication. But it’s getting better now. But I’m really nervous… that it will happen again.” MorganPT looked at Parker as they spoke, typed at times, paused when Parker finished, and simply said, “Ok” and moved on to ask about exercises. Although Parker spoke calmly and softly, Parker’s words clearly referenced their distress from an incident that caused severe pain and ongoing fear. Following suggestions from Pollak and Ashton-James (2018), this may have been an opportunity for MorganPT to empathise with Parker’s distress and acknowledge their experience by exploring some of the non-physical elements of Parker’s pain, but it was missed.

MorganPT employed questions to learn more about Parker’s concerns and goals. Parker repeatedly used words like “I cannot” and “I’m tired” to express their distress, but MorganPT did not acknowledge or affirm Parker’s distress before moving onto more questions. For example, when MorganPT was exploring Parker’s hopes from physio, Parker replied, “I do not know. Just relief from pain. I cannot do anything. If I do go out, when I get home, I have to sit down, I am so tired.” MorganPT quickly moved on by asking, “Before you had pain what were your activities like?” Parker replied, “I’m always doing something. I cannot lift my grandchildren anymore.”

This is the second time Parker refers to sleep or tiredness. The repeated allusion to tiredness could be a euphemism for depression or perhaps a more socially acceptable way of expressing it. Parker also referred to how their life used to be and expressed a sense of grief for no longer being able to lift grandchildren. Parker did not appear distressed in their tone of voice or body language, but their words and repetition indicated distress. This was left unacknowledged—or possibly even unrecognised—by MorganPT: a missed opportunity to connect, to let Parker know they heard and to investigate the socioemotional aspects of Parker’s pain. In moving on so quickly, MorganPT implicitly dismissed Parker’s story or deemed it irrelevant.

As MorganPT sought to wrap up the consult by organising a time for a follow-up appointment, Parker asked a series of questions. First about nerve pain and medication, specifically the medication (Tramadol—an opioid-like analgesic) they were taking. Parker explained, “I’m addicted. If I do not take it, I cannot sleep.” MorganPT, who was running 10 min late at this point, was focused on scheduling a follow-up appointment and replied that Parker did not need nerve pain medication and enquired, “If you are worried about Tramadol, have you got a good relationship with your GP?” MorganPT, possibly due to feeling under pressure to finish the consult, gave limited attention to the questions, deferred the question to the GP (which aligns with the scope of care), but also overlooked Parker’s concern surrounding these topics. Although Parker did not show concern regarding MorganPT’s distraction and deferral, Parker’s fear and distress could be contributing to their experience of pain.

Here, we could infer that MorganPT has adopted a classical psychology conceptualisation of distress as limited to universal physical presentations such as crying or a raised voice (Olson et al., 2020); perhaps, the reason they do not recognise or attend to Parker’s distress is because it is implicit and not expressed physically. Distress viewed through a physiological lens fails to recognise not only subtle expressions of distress, such as here through words and repetition but also sociocultural variances in how it might be expressed and that clinicians also experience distress (Dillon et al., Forthcoming). Covert meanings in words could be easily missed if a clinician is not attuned to what or how a patient is communicating. MorganPT’s lack of attunement to Parker’s words may also be a consequence of the distress and distraction they are bringing to the affective assemblage: late, sick child at home, with a limited capacity to be present and recognise their own distress or Parker’s. Unacknowledged and probably unrecognised, Parker’s distress was not explored or attended to with advice or management. This oversight may impact their engagement with treatment and pathway towards finding the appropriate strategies for understanding and managing their pain.

### Recognising and responding relationally

How physiotherapists recognise and respond to distress is not fixed—multiple social, emotional, cultural, personal, and systemic factors can shape how physiotherapists are able to grapple with distress each day in clinical encounters.

#### MorganPT and Quinn

While in the previous example, MorganPT likely did not recognise the patient’s distress, their first encounter with Quinn was different. They recognised Quinn’s distress and responded relationally throughout the interaction. Quinn’s distress was subtle, evident in their words and how he spoke. Although running late, MorganPT appeared calm and relaxed, began the encounter by apologising to Quinn and explained why they were behind. MorganPT sat in front of Quinn, seeming at ease, making eye contact, leaning towards them, and nodding as they spoke. Having reviewed Quinn’s notes in advance and knowing they had a scan recently, MorganPT suspected they may be worried. They immediately checked in to see if Quinn had received and understood the results. He sighed and softly replied: “Basically what they said from the CTscan: bulging discs, stenosis.” MorganPT repeated their words in a calm, soothing tone and slowly said, “the stenosis, yes, which is affecting the nerve roots that are coming down, and it looks like it’s affecting right and left equally.”

Being prepared meant MorganPT anticipated that Quinn might be distressed and attended to the topic immediately, possibly helping to alleviate their anxiety. Understanding the likely cause of distress perhaps gave MorganPT a sense of control over how to navigate the distress can be addressed. MorganPT’s open and receptive body language sitting down and leaning in, their vocal tone matching Quinn’s, may have signalled to them that they were listening intently. Practices such as “preparing with intention,” “listening with intent and completely,” “connecting with a patient’s story,” and “agreeing on what matters most,” have been suggested to help foster clinician presence and connection within clinical encounters (Zulman et al., 2020). MorganPT demonstrated many of these throughout the encounter.

Establishing what was important to Quinn, MorganPT enquired about their goals. Quinn explained softly, in a sad tone of voice, sighing and pausing at times, “I do not know what we can achieve. [pause] I can see something coming up that I am already worried about.” Quinn continued to explain that this was a work event where they would be required to stand for 3 days. MorganPT, nodding, maintaining eye contact, leaning towards Quinn, empathetically replied “yeah right,” but allowed them to continue. Quinn also explained how they can no longer play sport with their children. MorganPT clarified specific details regarding the type of sport and how pain was impacting it, finishing by summarising what they heard and offering Quinn an opportunity to correct or expand on their understanding.

Throughout the encounter, Quinn subtly expressed their distress in words and how they delivered them. MorganPT responded by giving them space to tell their story with minimal interruption, asking follow-up questions, gently seeking more details, and summarising what they heard, possibly indicating to Quinn that they were listening and interested in their story. This was in contrast to MorganPT’s previous observed encounter, where they appeared to be following a more scripted approach to questions, often not responding to the patient’s answer and moving on to the next topic. This possibly demonstrates how distress may impact a clinician’s ability to be flexible within encounters, falling back on checklists when distressed or uncomfortable.

Another subtle expression of distress was detected by MorganPT when screening for red flags, an important topic to ensure Quinn’s physical safety but a more sensitive subject. MorganPT enquired if anything had changed. Quinn hesitantly replied, “hmm no I do not think so, no.” MorganPT squinted their eyes, and Quinn started to laugh. MorganPT said firmly but kindly, “that did not convince me.” Quinn continued, “well I’ve noticed of late that I, I think it’s just the weather, that I feel like [hesitated] like I have to go to the toilet more.” MorganPT softly but confidently said, “ok, so increased urgency or frequency.” MorganPT detected Quinn’s discomfort and hesitancy, but rather than avoiding or ignoring it, they gently asked the necessary questions using their tone of voice and facial expressions to demonstrate that they understood them and cared, potentially helping Quinn to feel more at ease and share. It is probable that because MorganPT felt less distressed in this encounter, they were more able to be present and recognise Quinn’s distress and engage more relationally (Geller and Porges, 2014). MorganPT’s assertive but gentle tone possibly helped Quinn’s confidence in their care.

When physiotherapists consciously employ body language, tone of voice, pace, and question phrasing to foster presence and connection with their patients, it can serve to attend to the emotional dimensions of their patients’ pain experiences (Geller and Porges, 2014). In this encounter, such an approach acknowledged Quinn’s distress. It offered them the space to share their experiences, which offers insight into the socioemotional aspects of the pain-distress-assemblage. MorganPT guided the encounter by explaining what they were doing and why and was able to complete some of the checklist examinations but in a relational way that meant Quinn’s perspectives and emotions were understood, acknowledged, and attended to.

## Discussion

In this study, we adopted an affective assemblage approach to analysing physiotherapy care for people with CLBP—attending to the relational dimensions of the assemblage. Through this lens, it became clear that conceptualisations of distress matter to the clinical entanglement, particularly in how distress is recognised and navigated. Our findings indicate that distress is both a lived experience and an affective relation—that both the physiotherapist and people with CLBP experience distress and can be affected by and affect each other within clinical encounters. Situated at the intersection of health sociology, sociology of emotions, and physiotherapy, our study contributes to sociology with implications for physiotherapy practice.

Our study offers a worked example of the merits of applying an affective assemblage theoretical framework to understanding emotionally imbued clinical interactions. Construing distress as an affective assemblage pushes us to foreground emotion and the intersecting social forces imbuing the relationality of the clinical interaction for patients and clinicians. Without proposing a model or typology, this worked example of the value of adopting an affective assemblage framework offers sociologists an illustration of the epistemic and relational nuances in how physiotherapists attend to distress. Specifically, we identified various scripts—or professionally sanctioned ways of being and behaving—adopted by physiotherapists to navigate distress and clinical interactions.

Three scripts were identified in observations: cheer, biomedical, and protocol scripts. As seen in our study (Dillon et al., 2023), conforming to scripts can limit a clinician’s ability to attend to the socioemotional complexities of pain within clinical encounters. The scripts identified here position the clinician as the powerful expert and impose epistemic injustices on patients by privileging factual, objective knowledge and practices while marginalising the patient’s voice, their story, or how they think and feel about their pain. Such scripts do not just marginalise patients’ emotions.

Clinicians in our study reported how emotionally demanding CLBP care can be, with back-to-back technically difficult and often emotionally challenging encounters. Although some clinicians reported debriefing with colleagues following challenging encounters, no formal support structures were in place to care for clinicians. Expectations that clinicians adhere to cheer, biomedical, and professional protocol scripts cast clinicians as emotionally neutral (or at least only positive or superficial) and fail to recognise the affective and relational elements of care (Nicholls and Gibson, 2010; Setchell et al., 2019). Time-limited and scripted appointments constrain opportunities for compassion, empathic dialogue, and trusting therapeutic relationships. Such scripts give clinicians permission to leave some emotions—their own and their patients’—implicitly unacknowledged while encouraging others—perhaps especially in the case of the cheer script. In time-limited appointments, it is likely to promote surface acting; if repeated in back-to-back appointments, such surface acting may lead to unsatisfying encounters, burnout, and poor job satisfaction (Olson et al., 2017; Ashton-James et al., 2021).

Adopting an affective assemblage lens (Fox, 2015) sees varied scripts as part of the distress affective assemblage, affecting the clinician and patient and their capacities to act. It recognises the affective and relational aspects of care (Dragojlovic and Broom, 2018). It pushes for better systemic structures—and scripts—to be in place to care for patients and clinicians, allowing space for flexibility in practice and the prioritisation of more human aspects of care. Furthermore, calls have been made for institutional changes which provide the opportunity for “uncomfortable affectual experiences can be acknowledged, held and worked with” (Whittle et al., 2020, p. 10). Rather than sidelining clinician distress, the creation of these “safe spaces” allows recognition and acknowledgement at a system level that CLBP care can be distressing. These types of institutional changes can provide opportunities for reflexivity and the reframing of distress from an individual or bad experience, to a normal experience shared among clinicians when caring for people with CLBP (Whittle et al., 2020).

For physiotherapy practice, this study offers important implications. First, findings suggest that physiotherapy could benefit from reframing ways of understanding “safety.”

Current physiotherapy practices suggest that in applied usage at least, the meaning is limited to physical safety (Heywood et al., Forthcoming). They are acknowledging that distress is commonplace and an expected part of clinical encounters in a first step. Expanding the concept of safety to include emotional safety would encourage open recognition and acknowledgement of the distress and better training in how to attend to it (Miciak et al., 2018). It would help with the creation of emotionally safe spaces for consumers to talk about it and explore ways for the clinician to offer support (Plage et al., 2023).

In our study, distress circulated and fluctuated throughout all encounters. Patient’s distress was expressed in various ways, from subtle to more overt. Patient’s expressions of distress were uncomfortable for some physiotherapists, particularly when overt. Reasons for discomfort discussed in clinician dialogues included not knowing how to respond, fear for physical safety, or feeling threatened. Some clinicians described fear that patients’ overt expressions of distress can feel scary or abusive, particularly when a patient is expressing anger or frustration. Clinicians also described the pressure they experienced ensuring patients’ physical safety and not missing sinister pathology. This was particularly pertinent for clinical leaders working within a substitutionary model for surgical specialists. However, as illustrated through the data displays of MorganPT and Quinn’s clinical encounter, responding to distress can be as simple as being present or attentive, providing empathy, time, reassurance, and connection; it does not have to be complicated, but it may not be easy—especially if distress is excluded from models of care and safety. A new lens is needed.

Reframing distress as an affective assemblage (Dragojlovic and Broom, 2018) and expanding conceptualisations of safety to include emotional safety (Plage et al., 2023) may help clinicians to recognise patient distress as influenced by many things and reasonably considering the many challenges they face. Although aggression is unacceptable, such a reconceptualisation of distress and safety may support the recognition that such emotional expressions and their intensity are reasonable and part of a relational assemblage made up of clinicians’ and patients’ histories/past experiences, life challenges, and structural forces. Consumers in our consultatory panels, for example, suggested that it can be difficult to manage emotions, with many recounting experiences of crying in clinical encounters. They also described physiotherapists’ discomfort in such scenarios, with clinicians making jokes or changing the topic. Adopting an affective assemblage framework and expanding definitions of safety to include emotions would be the first step in working towards relational approaches to client-centred care.

A second would be recasting understandings of care beyond biomedical and narrow service-driven scopes to prioritise consumer-led definitions of care. Although the biopsychosocial model has attempted to move beyond the biomedical model, the focus on the body and rational approaches based on reductive forms of scientific approaches and technical fixes has unintentionally led to a neglect of other dimensions of the human experience like emotions—at times at the expense of human relationships (Sellman, 2012; Ahlsen et al., 2020). Adopting an affective assemblage framework not only broadens the scope of the biopsychosocial model but also shifts the focus beyond the individual in pain to include the physiotherapist and the many social, cultural, political, and structural forces that influence relation-centred CLBP care.

Shifting physiotherapy’s model of care, however, will be no small feat. This was a contentious topic within clinician dialogues. Some limited physiotherapy to purely exercise and physical approaches, deeming talking to a patient as insufficient. They expressed fear that “if I do not do this physical care, it does not count.” Some referred to emotions or non-physical elements of care as a “can of worms.” They feared not being able to navigate the moving chaos of the worms or that opening the can would exacerbate distress. Some physiotherapists suggest there are things that they cannot change, like a person’s socioeconomic status. They felt emphasis should be placed on how to achieve changes “quickly and easily” with specific exercises or deep breathing. Clinicians also highlighted the organisational challenges in shifting towards more encompassing and relational models of care: time-limited appointments, back-to-back emotionally and technically challenging patients, limited skill in knowing how to navigate emotions, outcome-driven service expectations, and team dynamics placing emphasis on technical and physical aspects of care. This points to the complexity of change and the many forces that influence physiotherapy practice, from individual preference and identity to historical, social, cultural, educational, and systemic forces.

Yet, as illustrated by our study, working within biomedical model-fostered scripted approaches that are task-orientated and transactional can be dehumanising and invalidating, limiting compassionate communication and the development of trust and safety within clinical encounters. These interpretations resonated with our consultatory panels. Mol’s (2008) contributions from her work on the logic of care can help to further the necessary shift in physiotherapy’s approach. Mol (2008) argued that care needs to be more than a transaction or an exchange; relating to others is an inextricable part of care. Mol (2008) argued that care is poor when there is no time to listen when physical factors are decontextualised or patients’ daily lives are not considered, when clinicians strictly follow protocols or scripts, and when the measurement of a few discrete parameters displaces attention from the intricacies of day-to-day life with disease or pain. Good care involves carefully applying evidence and delivering assessments and treatments with the patient’s experiences and lives in mind (Mol, 2008). The scientific tradition, which physiotherapy holds close, poorly accommodates unforeseen events or variables that cannot be counted, subjectivities, or timelines that do not follow a linear pattern. Care demands a more humanistic approach, where typical scripts will need, at times, to be tinkered with or abandoned (Dillon et al., 2023). Consumers in our consultatory panels emphasised the enormous impact of simply acknowledging and validating emotions and experiences. Thus, revisions to physiotherapy’s current model of care are needed.

It is important to acknowledge that this study—like all research—has limitations. It was conducted within a specialist service and a substitutionary model for surgical specialists within a large public hospital in Australia. While not all elements of the distress assemblage identified through our analysis will be the same in other healthcare contexts, such as a private practice, other cultural contexts, such as the Global South, or other conditions—the theoretical implications are likely transferable to several different contexts. However, this study has implications for future participatory research and for how reconceptualisations or improved understandings of emotions can impact CLBP care for both patients and clinicians. In addition, while the focus of this article was on the human aspects of distress in CLBP care, non-human and more than human factors merit scholarly attention in their own right and require further research. These include contextual factors such as the characteristics of the clinicians, patients, the therapeutic relationship, treatments, and the setting that can influence therapeutic outcomes (e.g., Cook et al., 2023).

## Conclusion

This article offers a worked example of the potential of applying affective assemblage theorisation to contexts at the intersection of health and emotional sociology. Viewing physiotherapy care through an affective assemblage lens allows for recognition that life, pain, and distress are emerging, always in flux. It renders problematic current biomedical protocol and cheers scripts that constrain flexibility and relationality and invites new and more affective approaches to care. Such an approach recognises that clinicians and patients experience distress; they are affected by and affect each other. Importantly, we are not suggesting neglecting the physicality of the body. What we do suggest is a shift away from the separation of the physical from emotional, mind from body, and carer from cared for.

## Data availability statement

The raw data supporting the conclusions of this article will be made available by the authors, without undue reservation.

## Ethics statement

The studies involving humans were approved by Dr. Gordon McGurk Human Research Ethics Committee, Royal Brisbane and Womens Hospital. The studies were conducted in accordance with the local legislation and institutional requirements. The participants provided their written informed consent to participate in this study.

## Author contributions

MD: Conceptualization, Data curation, Formal analysis, Investigation, Methodology, Writing – original draft. RO: Conceptualization, Formal analysis, Supervision, Writing – review & editing, Methodology. SP: Formal analysis, Supervision, Writing – review & editing, Conceptualization. MM: Formal analysis, Supervision, Writing – review & editing. PW: Formal analysis, Writing – review & editing. MS: Formal analysis, Writing – review & editing. AC: Formal analysis, Writing – review & editing. SK: Formal analysis, Writing – review & editing. NB: Formal analysis, Writing – review & editing. JS: Formal analysis, Methodology, Supervision, Writing – review & editing.

## Footnotes

^1^Relational theory considers that bodies, things, ideas, emotions, and social institutions do not exist independently but only through their relationship to the other (Fox, 2015).

^2^Assemblage is an interconnected network of sociomaterial forces (Barlott and Setchell, 2023).

^3^Scripts are set ways of thinking and doing that guide social interactions (Goffman, 1959).

^4^PT denotes multidisciplinary treating team physiotherapist within the clinic.

^5^Intentions to evince positive emotions may do the opposite (Setchell et al., 2019).

^6^Surface acting can be superficial or genuine, forced emotion management, reflecting attempts to change one’s displayed emotions (Hochschild, 2012).

^7^CL denotes Clinical Leader, advanced practice musculoskeletal physiotherapist.

^8^There was some similarity between RileyCL and AlexPT, both laughing at utterances of distress, with this laughter allowing them to segue into other things. However, the overall RileyCL’s overall approach was different, following a biomedical—rather than cheer—script.

^9^FrankiePT may be trying to understand what (physiologically) has prompted Blair’s distress—which aligns with physiotherapy training—rather than responding to distress itself.
