# Supplementary figures and images for: Distress in the care of people with chronic low back pain: insights from an ethnographic study
Source: Front Sociol. 2023 Nov 16;8:1281912. doi: 10.3389/fsoc.2023.1281912 (PMC10687466; doi:10.3389/fsoc.2023.1281912)

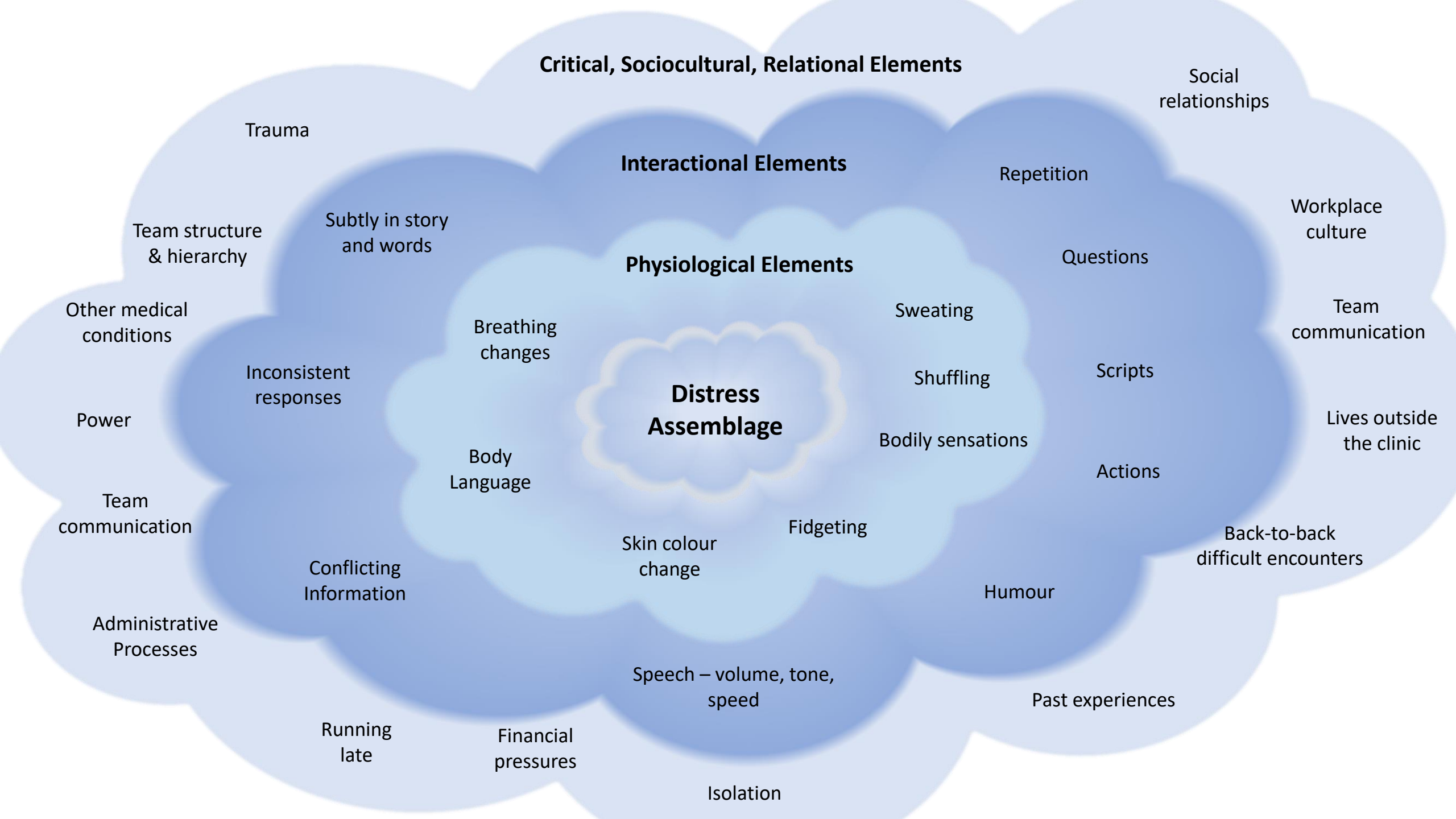

Supplement: Supplementary file 1 [file Data_Sheet_1.PDF]
